# Association between the dietary inflammatory index and all-cause mortality in osteoarthritis

**DOI:** 10.1186/s12891-024-07506-x

**Published:** 2024-05-23

**Authors:** Genglu Song, Yaoyu Lu

**Affiliations:** Department of Orthopedics and Traumatology, Qiannan Buyi and Miao Autonomous Prefecture Hospital of Traditional Chinese Medicine, No. 32 Jianjiang Middle Road, Qiannan Buyi and Miao Autonomous Prefecture, Duyun 558000 Guizhou, People’s Republic of China

**Keywords:** Dietary inflammatory index, All-cause mortality, Osteoarthritis, NHANES

## Abstract

**Background:**

To investigate the association between the Dietary Inflammatory Index (DII) and all-cause mortality in patients with osteoarthritis (OA).

**Methods:**

In this retrospective cohort study, data on OA patients were obtained from the National Health and Nutrition Examination Survey (NHANES) 2003–2018. OA diagnosis was self-reported. The study population was divided into low and high DII groups based on the DII’s median. All-cause mortality was the outcome, which was determined via linkage to the National Death Index (NDI) until 31 December 2019. Multivariable Cox regression analyses were employed to investigate the association between the DII and all-cause mortality. The survival of the low and high DII groups was exhibited by Kaplan–Meier curves. Furthermore, subgroup analyses were carried out in terms of age and comorbidity.

**Results:**

A total of 3804 patients with OA were included, with 1902 (50%) in the low DII group and 1902 (50%) in the high DII group. Patients with a high DII had a significantly greater risk of all-cause mortality than those with a low DII (HR = 1.21, 95%CI: 1.02–1.44, *P* = 0.025). A high DII was associated with a significantly increased risk of all-cause mortality compared with a low DII in patients aged ≥ 65 years [hazard ratio (HR) = 1.28, 95% confidence level (CI): 1.07–1.53, *P* = 0.006). Hypertensive patients with a high DII had a significantly greater risk of all-cause mortality than those with a low DII (HR = 1.25, 95%CI: 1.03–1.52, *P* = 0.025). For patients with cardiovascular disease (CVD), a high DII was associated with a significantly higher risk of all-cause mortality than a low DII (HR = 1.43, 95%CI: 1.17–1.75, *P* < 0.001). A high DII was associated with a significantly greater risk of all-cause mortality, as compared with a low DII in patients with chronic kidney disease (CKD) (HR = 1.22, 95%CI: 1.02–1.45, *P* = 0.026).

**Conclusion:**

The DII was positively associated with the risk of all-cause mortality in patients with OA. This association differed by age, hypertension, CVD, and CKD. Adherence to diet with a low DII may be beneficial in prognosis improvement.

**Supplementary Information:**

The online version contains supplementary material available at 10.1186/s12891-024-07506-x.

## Background

Osteoarthritis (OA) is a common chronic degenerative joint disorder, with rising incidence and prevalence related to age [1]. Approximately 7 000 out of every 100 000 people worldwide are affected by OA [2], and OA patients have a higher risk of all-cause and disease-specific mortality [3, 4]. This disease can affect different joints throughout the body, most commonly found in the knee, hip, and finger joints [5]. Patients with OA often experience joint pain, swelling and stiffness, symptoms worsen after prolonged activity, and OA, in severe cases, can lead to joint deformities and dysfunction, thus adversely impacting quality of life [6–8].

Chronic low-level systemic inflammation for a long term in the body can exacerbate the occurrence and progression of OA, and elevated levels of pro-inflammatory cytokines, such as tumor necrosis factor-α (TNF-α), interleukin-1β (IL-1β) and interleukin-6 (IL-6) have also been observed in the synovial fluid, synovium, and cartilage of OA patients, which will inhibit the synthesis of proteoglycans and type II collagen, and play a key role in the degradation of cartilage matrix and bone resorption in OA [9–11]. Dietary nutrition can regulate the chronic inflammatory state of the body to a certain extent [12]. Evidence shows that inflammatory diet is not only associated with the increased risk of chronic inflammatory diseases such as OA, cardiovascular disease (CVD) and diabetes, but also with the risk of all-cause and cause-specific death in CVD and diabetes [13–16]. Dietary intake itself, as a modifiable lifestyle, may also play an important role in the poor prognosis of OA. Based on this, quantitatively describing the inflammatory potential of an individual’s diet may have great potential in helping to predict outcomes of the OA population and improve prognosis through dietary interventions. Nowadays, there are various nutritional indices such as the Mediterranean diet index and the antioxidant index of the diet, which have been shown to be associated with osteoarthritis [17, 18]. The Dietary Inflammatory Index (DII), a literature-based dietary tool, was developed to evaluate the overall inflammatory potential of diet [19], including 45 food parameters related to inflammatory biomarkers [20]. Even though the DII is significantly associated with the increased risk of all-cause and cause-specific mortality of chronic inflammatory diseases such as CVD and diabetes [14, 16], it is unclear whether the DII is associated with the poor prognosis of OA patients.

This study aimed to investigate the association between the DII and all-cause mortality in patients with OA, using the data of the National Health and Nutrition Examination Survey (NHANES). This association was further explored in different age and comorbidity subpopulations.

## Methods

### Study population

In this retrospective cohort study, data on OA patients were obtained from 8 NHANES cycles (2003–2004, 2005–2006, 2007–2008, 2009–2010, 2011–2012, 2013–2014, 2015–2016, 2017–2018). The NHANES is a program of studies performed to investigate the health and nutritional status of the nationally representative population in the United States, which combines interviews and physical examinations to provide information about demographics, diets, physical examinations, laboratory tests, and questionnaire surveys [21]. The Ethics Review Board of the National Center for Health Statistics (NCHS) Research approves the NHANES survey. The requirement of ethical approval for this was waived by the Institutional Review Board of Qiannan Buyi and Miao Autonomous Prefecture Hospital of Traditional Chinese Medicine, because the data was accessed from NHANES (a publicly available database). The need for written informed consent was waived by the Institutional Review Board of Qiannan Buyi and Miao Autonomous Prefecture Hospital of Traditional Chinese Medicine due to retrospective nature of the study. All methods were performed in accordance with the relevant guidelines and regulations. Patients diagnosed with OA were included in the current study. Patients with missing survival data, aged less than 20 years, with missing dietary data required for DII calculation, with extreme values of energy intake (< 500 or > 8000 kcal for males; < 500 or > 5000 kcal for females), and with missing data on body mass index (BMI), age, sex, and neutrophil-to-lymphocyte ratio (NLR) were ruled out. Eligible patients were followed up until December 31, 2019.

## OA assessment

OA diagnosis was self-reported. Self-reported OA was reported to have high consistency (85%) with clinically confirmed OA [13]. For the question “Has a doctor or other health professional ever told you that you had arthritis?”, a positive response was followed by another question “Which type of arthritis was it?”. Based on the response to the latter question, subjects could be considered to have OA.

## DII calculation

The DII was calculated using the approach developed by Shivappa et al. [20]. First, a Z score was obtained by subtracting the global average daily intake of a dietary component from the average daily intake of the dietary component, followed by division by the standard deviation. Second, the obtained score was converted to a percentile value, doubled, and then subtracted by 1. Subsequently, the value obtained from the second step was multiplied by the inflammatory effect score of the dietary component. Finally, a DII score was obtained by dividing by energy and multiplying by 1000. An individual’s overall DII score was calculated by summing the DII score of each dietary component. According to the total nutrient intakes from the dietary interview on the first day, 27 nutrients were utilized for DII calculation in this study, including saturated fat, total fat, energy, cholesterol, carbohydrate, ferrum, protein, vitamins B1, B2, B6 and B12, thyme, caffeine, folic acid, selenium, alcohol, zinc, polyunsaturated fatty acid (PUFA), monounsaturated fatty acid (MUFA), vitamins A, E and C, omega-3 fatty acids, omega-6 fatty acids, magnesium, β-carotene, and cellulose. A higher DII score indicated a greater level of dietary inflammation. Then the study population was divided into low (DII < 0.7705) and high DII (DII ≥ 0.7705) groups based on the DII’s median (0.7705).

## Mortality assessment and other variables

All-cause mortality was the outcome of this study, which was determined via linkage to the National Death Index (NDI) until 31 December 2019 (https://www.cdc.gov/nchs/data-linkage/mortality.htm). Information on the following variables was also collected: age (years), sex, race (non-Hispanic black, non-Hispanic white, and other), BMI (kg/m^2^), poverty income ratio (PIR) (low/median, high), marital status (married/living with partner, divorced/widowed/separated, spinsterhood), education [below high school, high school/general education development (GED), college and above, some college/associate (AA) degree], smoking (no, yes), drinking (no, yes, unknown), hypertension (no, yes), diabetes (no, yes), dyslipidemia (no, yes), CVD (no, yes), chronic obstructive pulmonary disease (COPD) (no, yes), depression (no, yes, unknown), cancer (no, yes), chronic kidney disease (CKD) (no, yes), drug therapy (no, yes), systolic blood pressure (SBP, mmHg), diastolic blood pressure (DBP, mmHg), physical activity (mild, median/strenuous, unknown), glycohemoglobin (%), total cholesterol (TC, mmol/L), high-density lipoprotein (HDL, mmol/L), alanine transaminase/aspartate transaminase (ALT/AST), albumin/globulin (A/G), NLR, estimated glomerular filtration rate (eGFR), urinary albumin creatinine ratio (UACR, mg/g), total energy, and macronutrients and a number of important nutrients used in the DII calculation. Physical activity was presented as energy consumption (MET·min), which was calculated by multiplying recommended metabolic equivalent (MET) by exercise time corresponding to the activity (min). Physical activity < 450 and ≥ 450 MET·min was classified as mild and median/strenuous, respectively. Drug therapy was defined as use of non-steroidal drugs and salicylate drugs.

## Statistical analysis

Measurement data were described as mean (standard error) [Mean (SE)], and weighted analysis of variance was used for comparisons between the two groups. Enumeration data were reported in the number of cases and percentage [n (%)], inter-group comparisons were performed using the Rao-Scott Chi-square test. Variables with missing data accounting for less than 10% were filled in using random forest imputation, and differences between pre- and post-imputation data were analyzed (Supplementary Table 1). If the proportion of missing data exceeded 10%, the variables would be processed into categorical data. Samples with missing data exceeding 20% were excluded.

Statistically significant variables in the weighted univariable Cox regression model were taken as potential confounding factors (Supplementary Table 2). All potential confounding factors were incorporated into the multivariable Cox regression model, and the stepwise backward screening method was used to select all statistically significant confounding factors in the multivariable Cox model as final covariables (Supplementary Table 3), in order to correct for the association between the DII and all-cause mortality in OA patients. Univariable and multivariable Cox regression analyses were employed to investigate the association between the DII and all-cause mortality, and in the adjusted model, age, sex, marital status, smoking, hypertension, CVD, depression, CKD, ALT/AST, A/G, and NLR were adjusted for. Considering the close relationship between BMI and DII, BMI was further adjusted for in the model. The survival of the low and high DII groups was exhibited by Kaplan–Meier curves and compared using the log-rank test. Furthermore, subgroup analyses were carried out in terms of age (< 65, ≥ 65 years) and comorbidity (hypertension, CVD, CKD) by using multivariable regression to assess whether the risk of all-cause mortality differed in different OA subpopulations. Hazard ratios (HRs) and 95% confidence levels (CIs) were estimated.

Data cleaning (including missing value statistics), missing value imputation, statistical modeling, and subgroup analyses were completed using R 4.2.2 (R Foundation for Statistical Computing, Vienna, Austria). Data extraction, sensitivity analysis, and descriptive statistics were performed by SAS 9.4 (SAS Institute Inc., Cary, NC, USA). Differences were regarded statistically significant when *P* values were less than 0.05.

## Results

### Characteristics of the study population

**Fig. 1:**
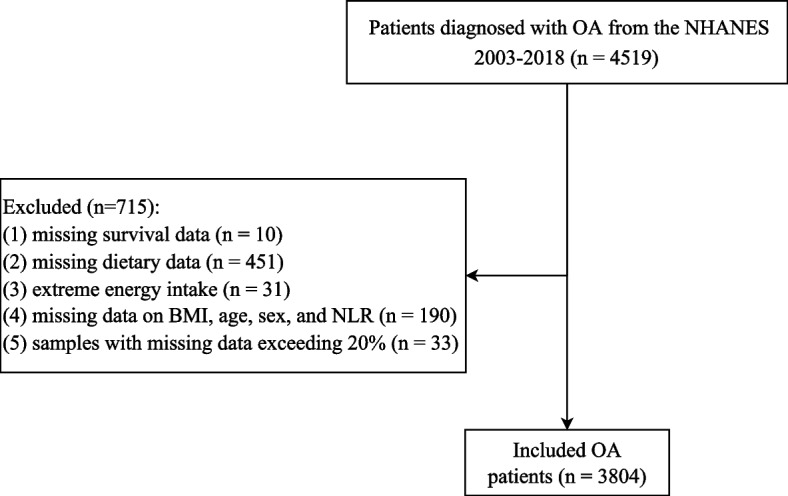
Flow chart of participant selection. OA, osteoarthritis; BMI, body mass index; NLR, neutrophil-to-lymphocyte ratio

A total of 4519 patients with OA were enrolled from the NHANES 2003–2018. After excluding patients with missing survival data (*n* = 10), with missing dietary data required for DII calculation (*n* = 451), with extreme values of energy intake (< 500 or > 8000 kcal for males; < 500 or > 5000 kcal for females) (*n* = 31), and with missing data on BMI, age, sex, and NLR (*n* = 10), and samples with missing data exceeding 20% (*n* = 33), 3804 patients were eventually included, with 1902 (50%) in the low DII group and 1902 (50%) in the high DII group. Figure 1 presents the flow chart of participant selection. The average age of these patients with OA was 61.81 years. The high DII group had significantly higher proportions of females (75.40% vs 55.21%), low/median PIRs (52.20% vs 38.46%), divorced/widowed/separated marital status (24.80% vs 32.80%), CVD (37.63% vs 31.62%), and COPD (34.56% vs 27.31%) than the low DII group (all *P* < 0.05). Table 1 shows the detailed characteristics of the included patients with OA.

**Table 1:** Characteristics of the included patients with OA

Variables	Total (*n* = 3804)	Low DII group (*n* = 1902)	High DII group (*n* = 1902)	Statistics	*P*
**Age, years, Mean (SE)**	61.81 (0.31)	61.45 (0.41)	62.24 (0.46)	t = 1.319	0.190
**Sex, n (%)**				χ^2^ = 101.526	< 0.001
Female	2420 (64.60)	1026 (55.21)	1394 (75.40)		
Male	1384 (35.40)	876 (44.79)	508 (24.60)		
**Race, n (%)**				χ^2^ = 7.189	0.003
Non-Hispanic black	556 (6.12)	251 (4.93)	305 (7.49)		
Non-Hispanic white	2466 (83.27)	1288 (85.78)	1178 (80.39)		
Other	782 (10.61)	363 (9.29)	419 (12.11)		
**BMI, kg/m**^**2**^**, Mean (SE)**	30.72 (0.18)	30.54 (0.22)	30.92 (0.25)	t = 1.267	0.208
**PIR, n (%)**				χ^2^ = 41.213	< 0.001
Low/median	2204 (44.85)	975 (38.46)	1229 (52.20)		
High	1600 (55.15)	927 (61.54)	673 (47.80)		
**Marital status, n (%)**				χ^2^ = 6.954	0.002
Married/living with partner	2239 (64.13)	1210 (67.50)	1029 (60.25)		
Divorced/widowed/separated	1303 (28.52)	565 (24.80)	738 (32.80)		
Spinsterhood	262 (7.35)	127 (7.70)	135 (6.96)		
**Education, n (%)**				χ^2^ = 22.064	< 0.001
Below high school	686 (11.62)	259 (9.72)	427 (13.80)		
High school/GED	942 (24.70)	435 (20.83)	507 (29.14)		
College and above	1219 (32.92)	611 (31.84)	608 (34.16)		
Some college/AA degree	957 (30.77)	597 (37.62)	360 (22.90)		
**Smoking, n (%)**				χ^2^ = 0.007	0.935
No	1796 (47.22)	883 (47.31)	913 (47.12)		
Yes	2008 (52.78)	1019 (52.69)	989 (52.88)		
**Drinking, n (%)**				χ^2^ = 9.328	< 0.001
No	971 (21.55)	407 (17.30)	564 (26.44)		
Yes	2151 (59.06)	1146 (62.23)	1005 (55.42)		
Unknown	682 (19.39)	349 (20.48)	333 (18.15)		
**Hypertension, n (%)**				χ^2^ = 0.875	0.351
No	1057 (33.01)	561 (34.03)	496 (31.85)		
Yes	2747 (66.99)	1341 (65.97)	1406 (68.15)		
**Diabetes, n (%)**				χ^2^ = 2.791	0.097
No	1954 (55.50)	1027 (57.20)	927 (53.54)		
Yes	1850 (44.50)	875 (42.80)	975 (46.46)		
**Dyslipidemia, n (%)**				χ^2^ = 1.267	0.263
No	762 (19.75)	408 (20.61)	354 (18.75)		
Yes	3042 (80.25)	1494 (79.39)	1548 (81.25)		
**CVD, n (%)**				χ^2^ = 9.770	0.002
No	2339 (65.58)	1226 (68.38)	1113 (62.37)		
Yes	1465 (34.42)	676 (31.62)	789 (37.63)		
**COPD, n (%)**				χ^2^ = 14.058	< 0.001
No	2578 (69.32)	1342 (72.69)	1236 (65.44)		
Yes	1226 (30.68)	560 (27.31)	666 (34.56)		
**Depression, n (%)**				χ^2^ = 13.428	< 0.001
No	2294 (61.38)	1221 (65.93)	1073 (56.14)		
Yes	1106 (29.49)	491 (25.88)	615 (33.64)		
Unknown	404 (9.13)	190 (8.19)	214 (10.22)		
**Cancer, n (%)**				χ^2^ = 0.564	0.454
No	2973 (77.02)	1477 (77.72)	1496 (76.21)		
Yes	831 (22.98)	425 (22.28)	406 (23.79)		
**CKD, n (%)**				χ^2^ = 0.810	0.370
No	283 (7.16)	136 (6.81)	147 (7.57)		
Yes	3521 (92.84)	1766 (93.19)	1755 (92.43)		
**Drug therapy, n (%)**				χ^2^ = 1.719	0.192
No	3263 (85.93)	1667 (87.01)	1596 (84.68)		
Yes	541 (14.07)	235 (12.99)	306 (15.32)		
**SBP, mmHg, Mean (SE)**	128.03 (0.45)	127.74 (0.60)	128.36 (0.61)	t = 0.747	0.457
**DBP, mmHg, Mean (SE)**	70.44 (0.32)	71.24 (0.42)	69.53 (0.39)	t = -3.450	0.001
**Physical activity, n (%)**				χ^2^ = 26.041	< 0.001
Mild	2301 (56.95)	1061 (50.91)	1240 (63.89)		
Median/strenuous	1171 (35.63)	708 (42.91)	463 (27.26)		
Unknown	332 (7.42)	133 (6.18)	199 (8.85)		
**Glycohemoglobin, %, Mean (SE)**	5.79 (0.02)	5.74 (0.02)	5.83 (0.03)	t = 2.556	0.012
**TC, mmol/L, Mean (S.E)**	5.14 (0.02)	5.09 (0.03)	5.20 (0.03)	t = 2.649	0.009
**HDL, mmol/L, Mean (S.E)**	1.44 (0.01)	1.43 (0.01)	1.44 (0.02)	t = 0.493	0.623
**ALT/AST, Mean (SE)**	0.94 (0.01)	0.97 (0.01)	0.90 (0.01)	t = -4.862	< 0.001
**A/G, Mean (SE)**	1.55 (0.01)	1.59 (0.01)	1.51 (0.01)	t = -5.324	< 0.001
**NLR, Mean (SE)**	2.38 (0.03)	2.39 (0.04)	2.37 (0.04)	t = -0.494	0.622
**eGFR, mL/min/1.73 m**^**2**^**, Mean (SE)**	77.26 (0.46)	78.36 (0.58)	75.98 (0.66)	t = -2.845	0.005
**UACR, mg/g, Mean (SE)**	2814.15 (276.88)	2410.03 (243.74)	3278.85 (499.16)	t = 1.624	0.107
**DII, Mean (SE)**	0.94 (0.04)	-0.17 (0.02)	2.22 (0.04)	t = 50.012	< 0.001
**Total energy, kcal, Mean (SE)**	1953.30 (18.19)	2372.31 (24.84)	1471.48 (17.96)	t = -29.140	< 0.001
**Protein, g, Mean (SE)**	75.95 (0.95)	93.86 (1.33)	55.35 (0.79)	t = -24.867	< 0.001
**Carbohydrate, g, Mean (SE)**	232.17 (2.33)	276.74 (3.68)	180.91 (2.69)	t = -20.617	< 0.001
**Total fat, g, Mean (SE)**	77.48 (0.99)	95.28 (1.48)	57.01 (0.84)	t = -21.023	< 0.001
**TSFA, g, Mean (SE)**	25.48 (0.39)	30.40 (0.64)	19.82 (0.35)	t = -14.039	< 0.001
**TMUFA, g, Mean (SE)**	27.50 (0.37)	33.98 (0.55)	20.04 (0.32)	t = -20.676	< 0.001
**TPUFA, g, Mean (SE)**	17.65 (0.27)	22.51 (0.37)	12.07 (0.23)	t = -23.127	< 0.001
**Cholesterol, mg, Mean (SE)**	265.88 (4.68)	317.45 (8.00)	206.57 (4.81)	t = -11.484	< 0.001
**Dietary fiber, g, Mean (SE)**	16.51 (0.28)	21.78 (0.38)	10.45 (0.15)	t = -30.427	< 0.001
**Vitamin A, mcg, Mean (SE)**	639.96 (12.84)	867.23 (19.53)	378.63 (7.90)	t = -24.618	< 0.001
**Octadecatrienoic, g, Mean (SE)**	1.67 (0.04)	2.15 (0.05)	1.12 (0.03)	t = -18.721	< 0.001
**Octadecatetraenoic, g, Mean (SE)**	0.01 (0.00)	0.01 (0.00)	0.01 (0.00)	t = -3.610	< 0.001
**Eicosapentaenoic, g, Mean (SE)**	0.03 (0.00)	0.05 (0.00)	0.02 (0.00)	t = -6.949	< 0.001
**Docosapentaenoic, g, Mean (SE)**	0.02 (0.00)	0.03 (0.00)	0.01 (0.00)	t = -9.696	< 0.001
**Docosahexaenoic, g, Mean (SE)**	0.07 (0.00)	0.09 (0.01)	0.04 (0.00)	t = -8.775	< 0.001
**Octadecadienoic, g, Mean (SE)**	15.55 (0.24)	19.81 (0.34)	10.65 (0.21)	t = -22.617	< 0.001
**Eicosatetraenoic, g, Mean (SE)**	0.13 (0.00)	0.16 (0.00)	0.10 (0.00)	t = -10.727	< 0.001
**β-Carotene, mcg, Mean (SE)**	2324.42 (102.29)	3423.11 (169.29)	1061.05 (59.23)	t = -13.581	< 0.001
**Thiamine, mg, Mean (SE)**	1.54 (0.02)	1.93 (0.03)	1.10 (0.02)	t = -24.067	< 0.001
**Riboflavin, mg, Mean (SE)**	2.15 (0.04)	2.67 (0.06)	1.56 (0.03)	t = -17.283	< 0.001
**Niacin, mg, Mean (SE)**	23.58 (0.43)	29.70 (0.63)	16.55 (0.33)	t = -18.859	< 0.001
**Vitamin B6, mg, Mean (SE)**	1.96 (0.04)	2.55 (0.06)	1.29 (0.05)	t = -16.218	< 0.001
**Total folate, mcg, Mean (SE)**	385.39 (5.19)	496.29 (7.91)	257.86 (4.06)	t = -25.821	< 0.001
**Vitamin B12, mcg, Mean (SE)**	4.89 (0.13)	6.37 (0.21)	3.19 (0.09)	t = -14.086	< 0.001
**Vitamin C, g, Mean (SE)**	81.90 (1.89)	110.32 (2.61)	49.22 (1.51)	t = -21.914	< 0.001
**Vitamin E, mg, Mean (SE)**	8.25 (0.13)	11.19 (0.17)	4.87 (0.08)	t = -33.939	< 0.001
**Magnesium, mg, Mean (SE)**	291.83 (3.60)	373.42 (4.30)	198.01 (2.35)	t = -36.416	< 0.001
**Ferrum, mg, Mean (SE)**	14.33 (0.18)	18.27 (0.29)	9.79 (0.13)	t = -26.069	< 0.001
**Zinc, mg, Mean (SE)**	10.88 (0.14)	13.71 (0.21)	7.62 (0.14)	t = -22.996	< 0.001
**Selenium, mcg, Mean (SE)**	103.88 (1.30)	127.73 (2.01)	76.45 (1.19)	t = -20.828	< 0.001
**Caffeine, mg, Mean (SE)**	200.85 (6.47)	208.63 (6.98)	191.91 (9.17)	t = -1.734	0.085
**Alcohol, g, Mean (SE)**	7.59 (0.47)	10.17 (0.77)	4.63 (0.49)	t = -6.106	< 0.001

*OA* osteoarthritis, *DII* National Death Index, *SE* standard error, *BMI* body mass index, *PIR* poverty income ratio, *GED* general education development, *AA* associate, *CVD* cardiovascular disease, *COPD* obstructive pulmonary disease, *CKD* chronic kidney disease, *SBP* systolic blood pressure, *DBP* diastolic blood pressure, *TC* total cholesterol, *HDL* high-density lipoprotein, *ALT/AST* alanine transaminase/aspartate transaminase, *A/G* albumin/globulin, *NLR* neutrophil-to-lymphocyte ratio, *eGFR* estimated glomerular filtration rate, *UACR* urinary albumin creatinine ratio

### Association between the DII and all-cause mortality in OA

**Fig. 2:**
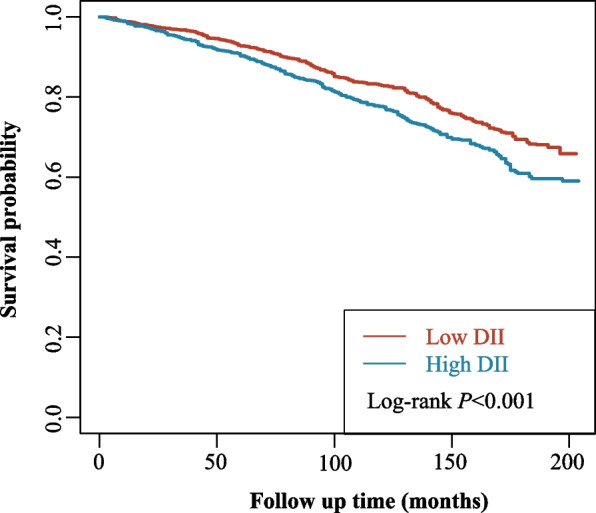
Kaplan–Meier survival curves of the low and high DII groups. DII, Dietary Inflammatory Index

After adjusting for age, sex, marital status, smoking, hypertension, CVD, depression, CKD, physical activity, ALT/AST, A/G, NLR, and BMI, patients with a high DII had a significantly greater risk of all-cause mortality than those with a low DII (HR = 1.21, 95%CI: 1.02–1.44, *P* = 0.025) (Table 2). As shown by Fig. 2, a high DII was associated with significantly worse survival compared with a low DII (log-rank *P* < 0.001).

**Table 2:** Association between the DII and all-cause mortality in OA

Variables	Unadjusted		Model 1		Model 2	
	**HR (95% CI)**	***P***	**HR (95% CI)**	***P***	**HR (95% CI)**	***P***
**Group**						
Low DII	Ref		Ref		Ref	
High DII	1.36 (1.15–1.60)	< 0.001	1.21 (1.02–1.44)	0.025	1.21 (1.02–1.44)	0.025

Model 1, a multivariable model, adjusted for age, sex, marital status, smoking, hypertension, CVD, depression, CKD, physical activity, ALT/AST, A/G, and NLR

Model 2, a multivariable model, adjusted for age, sex, marital status, smoking, hypertension, CVD, depression, CKD, physical activity, ALT/AST, A/G, NLR, and BMI

*OA* osteoarthritis, *DII* National Death Index, *CVD* cardiovascular disease, *CKD* chronic kidney disease, *ALT/AST* alanine transaminase/aspartate transaminase, *A/G* albumin/globulin, *NLR* neutrophil-to-lymphocyte ratio, *BMI* body mass index, *HR* hazard ratio, *CI* confidence level, *Ref* reference

### Association between the DII and all-cause mortality stratified by age and comorbidity

#### Age

**Fig. 3:**
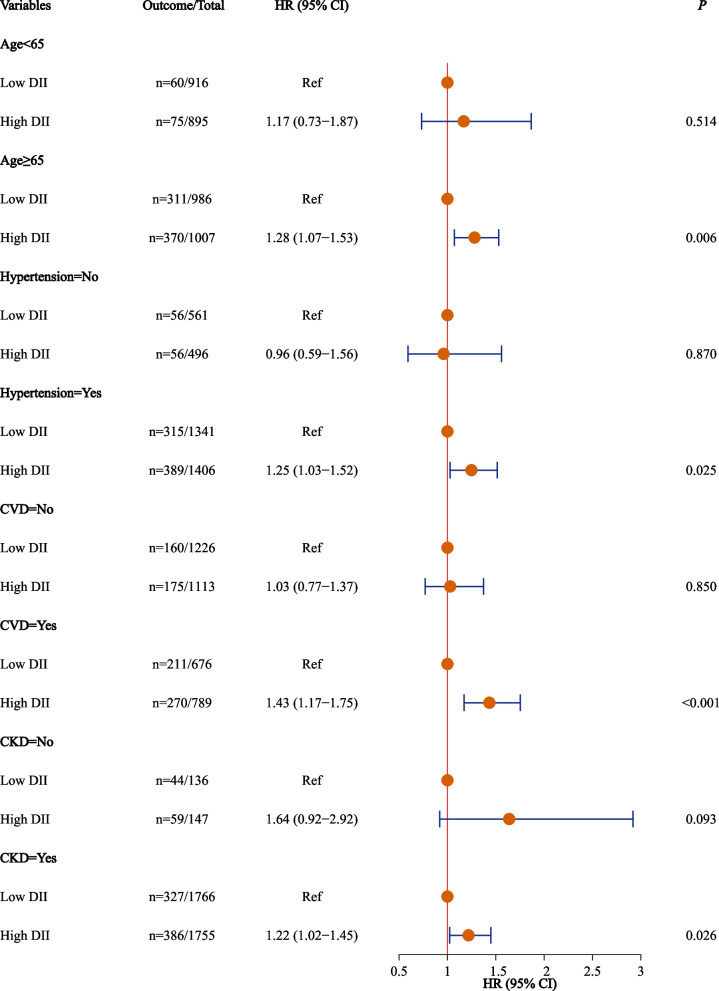
Association between the DII and all-cause mortality stratified by age and comorbidity. For age < 65 years subgroup: age, sex, race, smoking, hypertension, COPD, A/G, NLR, and BMI; For age ≥ 65 years subgroup: age, sex, marital status, smoking, hypertension, CVD, depression, CKD, physical activity, ALT/AST, A/G, NLR, and BMI; For no hypertension subgroup: age, marital status, smoking, cancer, A/G, NLR, and BMI; For hypertension subgroup: age, sex, marital status, CVD, depression, CKD, physical activity, ALT/AST, A/G, NLR, and BMI; For no CVD subgroup: age, smoking, hypertension, depression, physical activity, NLR, and BMI; For CVD subgroup: age, sex, marital status, hypertension, physical activity, ALT/AST, A/G, NLR, and BMI; For no CKD subgroup: age, sex, physical activity, and BMI; For CKD subgroup: age, sex, education, hypertension, physical activity, ALT/AST, NLR, and BMI. DII, National Death Index; CVD, cardiovascular disease; CKD, chronic kidney disease; ALT/AST, alanine transaminase/aspartate transaminase; A/G, albumin/globulin; NLR, neutrophil-to-lymphocyte ratio; BMI, body mass index; HR, hazard ratio; CI, confidence level; Ref, reference

A high DII was associated with a significantly increased risk of all-cause mortality compared with a low DII in patients aged ≥ 65 years (HR = 1.28, 95%CI: 1.07–1.53, *P* = 0.006) (Fig. 3).

#### Comorbidity

##### Hypertension

Hypertensive patients with a high DII had a significantly greater risk of all-cause mortality than those with a low DII (HR = 1.25, 95%CI: 1.03–1.52, *P* = 0.025) (Fig. 3).

**CVD**

For patients with CVD, a high DII was associated with a significantly higher risk of all-cause mortality than a low DII (HR = 1.43, 95%CI: 1.17–1.75, *P* < 0.001) (Fig. 3).

**CKD**

A high DII was associated with a significantly greater risk of all-cause mortality, as compared with a low DII in patients with CKD (HR = 1.22, 95%CI: 1.02–1.45, *P* = 0.026) (Fig. 3).

## Discussion

Mounting evidence suggests that inflammation plays an essential role in the pathophysiological progression of OA [22, 23]. Whether the inflammatory potential of diet, a modifiable factor, is related to the prognosis of patients with OA needs assessment. This study probed into the association between the DII and all-cause mortality in patients with OA for the first time, and found a positive association between the DII and the risk of all-cause mortality in OA. Furthermore, this association varied by age, hypertension, CVD, and CKD. These findings suggested that in the management and treatment of OA, paying attention to and improving patients’ dietary patterns, particularly by adjusting the diet to reduce inflammatory levels (choosing anti-inflammatory foods, and reducing the intake of pro-inflammatory foods), may play a significant role in improving prognosis and prolonging survival. Physicians and dietitians may also need to tailor dietary interventions based on individual patient characteristics, such as age and the presence of hypertension, CVD and CKD, in order to better improve patient outcomes.

For individuals with OA, a higher DII was associated with a greater risk of all-cause mortality in the present study, suggesting that an anti-inflammatory dietary pattern may help improve the prognosis of OA patients. As shown by Shivappa et al. [14], a higher DII was associated with an elevated risk of CVD risk and related mortality. The association between the DII and all-cause mortality was demonstrated by several studies [16, 24–26]. Cao et al. [27] illustrated those elderly hypertensive patients with a lower DII had a reduced risk of all-cause death. The DII investigates the inflammatory contribution of diverse dietary components [20], and recognizes the food matrix or the complex interactions of dietary components within foods and dietary patterns [24]. The bioactive compounds considered in this study may account for the association identified between the DII and all-cause mortality in OA. Among the evaluated food parameters, saturated fat, total fat, energy, cholesterol, carbohydrate, ferrum, protein, and vitamin B12 exhibited pro-inflammatory properties. Saturated fat may contribute to inflammation, for example, through toll-like receptor 4 expression and gut microbiota regulation [28, 29]. The potential of omega-3 fatty acids to reduce inflammation has been extensively researched, which may involve modified phospholipid fatty acid composition of cell membranes, disrupted lipid rafts, suppressed pro-inflammatory factor κB activation, and so forth [30, 31]. Cholesterol is actively metabolized and can modulate several aspects of inflammation by its mobilization and/or generation of active derivatives [32]. The anti- and pro-inflammatory impacts of food components appear to be mediated by gut microbiota as well [33]. OA was reported to be linked to joint pain, activity restriction, physical impairment, decreased health-related quality of life, and an increased risk of death [34], which may be facilitated by pro-inflammatory diet with a high DII.

Furthermore, the association between the DII and all-cause mortality varied in OA subpopulations in different age, hypertension, CVD, and CKD groups. For older adults, increased levels of inflammation raise the risk of poor health and death [35]. Any extra inflammation may exceed a tolerable threshold in the setting of an already high inflammatory state owing to inflammageing, resulting in substantial tissue damage, illness, and even death [35]. Thus, OA patients aged ≥ 65 years who had diet with a higher DII may at an elevated risk of all-cause mortality. Notably, as regards individuals with inflammatory diseases, including hypertension, CVD and CKD [36], an anti-inflammatory dietary pattern may lower the risk of all-cause death. Patients with CKD typically exhibit an increased systemic inflammatory response, which may arise from a variety of factors such as the accumulation of metabolic products due to renal insufficiency and dysregulation of the immune system. A DII score indicates that dietary habits are more inclined towards promoting inflammation within the body. This additional inflammatory state may be superimposed upon the existing inflammatory foundation in CKD patients, which may exacerbate the systemic inflammatory condition, consequently impacting overall survival. These specific patients with OA are supposed to focus on the overall inflammatory potential of their diet, and make corresponding adjustments when necessary, so as to improve their prognosis.

This study utilized a nationally representative sample from the NHANES database, and first investigated the association between the DII and all-cause mortality in OA. Based on the findings, following an anti-inflammatory dietary pattern might be advantageous to prognosis improvement. For example, patients with OA can choose foods rich in vitamins A, E, C, B1, B2, and B6, unsaturated fatty acids, and β-carotene, so that favorable outcomes may be achieved. Several limitations should be recognized: (1) the study participants may change their eating habits due to the diagnosis of certain diseases (such as diabetes and CVD), so the collected dietary information may not represent the daily eating habits of the participants; (2) the calculation of DII only considered the first 24-h dietary interview data of participants, and the estimated individual dietary intake did not take into account daily changes in diet or seasonal changes in dietary patterns. Besides, although 45 foods and nutrients were used to calculate the inflammatory index of the diet based on the Shivapa method, 27 nutrients were utilized for DII calculation in this study since data on other 18 studies were not available in the NHANES database; (3) although many covariables were included in the model, the confounding effects caused by unmeasured or unavailable covariables (such as the severity of OA, CRP level, etc.) could not be ruled out. Future studies are necessitated to consider the above factors to improve the research on the association between the DII and all-cause mortality in individuals with OA.

## Conclusion

A higher DII was associated with a greater risk of all-cause mortality in patients with OA. This association remained significant in OA patients aged ≥ 65 years, and with hypertension, diabetes, CVD, and depression. These patients may benefit from adherence to diet with a low DII. These findings need to be verified by more studies.

### Supplementary Information

Supplementary file 1.

## Data Availability

The datasets generated during and/or analyzed during the current study are available in the NHANES database, https://www.cdc.gov/nchs/nhanes/index.htm.

## Abbreviations

OA: Osteoarthritis
TNF-α: Tumor necrosis factor-α
IL-1β: Interleukin-1β
IL-6: Interleukin-6
CVD: Cardiovascular disease
DII: Dietary Inflammatory Index
NHANES: National Health and Nutrition Examination Survey
PUFA: Polyunsaturated fatty acid
MUFA: Monounsaturated fatty acid
NDI: National Death Index
PIR: Poverty income ratio
GED: General education development
AA: V associate
COPD: Chronic obstructive pulmonary disease
CKD: Chronic kidney disease
SBP: Systolic blood pressure
DBP: Diastolic blood pressure
TC: Total cholesterol
HDL: High-density lipoprotein
ALT/AST: Alanine transaminase/aspartate transaminase
A/G: Albumin/globulin
eGFR: Estimated glomerular filtration rate
UACR: Urinary albumin creatinine ratio;
MET: Metabolic equivalent
