# Supplementary material for: Association between the dietary inflammatory index and all-cause mortality in osteoarthritis
Source: BMC Musculoskelet Disord. 2024 May 23;25:407. doi: 10.1186/s12891-024-07506-x (PMC11112835; doi:10.1186/s12891-024-07506-x)
Supplement: Supplementary file 1 [file 12891_2024_7506_MOESM1_ESM.docx]

Supplementary Table 1 Differences between pre- and post-imputation data.

| **Variables** | **Pre-imputation (n=3804)** | **Post-imputation (n=3804)** | **Statistics** | ***P*** |
| --- | --- | --- | --- | --- |
| **PIR, n (%)** |  |  | χ^2^=0.461 | 0.497 |
| Low/median | 2021 (57.15) | 2204 (57.94) |  |  |
| High | 1515 (42.85) | 1600 (42.06) |  |  |
| **Marital status, n (%)** |  |  | χ^2^=0.000 | 1.000 |
| Married/living with partner | 2238 (58.85) | 2239 (58.86) |  |  |
| Divorced/widowed/separated | 1303 (34.26) | 1303 (34.25) |  |  |
| Spinsterhood | 262 (6.89) | 262 (6.89) |  |  |
| **Education, n (%)** |  |  | χ^2^=2.312 | 0.510 |
| Below high school | 617 (16.93) | 686 (18.03) |  |  |
| High school/GED | 888 (24.36) | 942 (24.76) |  |  |
| College and above | 954 (26.17) | 957 (25.16) |  |  |
| Some college/AA degree | 1186 (32.54) | 1219 (32.05) |  |  |
| **Smoking, n (%)** |  |  | χ^2^=0.000 | 0.990 |
| No | 1795 (47.20) | 1796 (47.21) |  |  |
| Yes | 2008 (52.80) | 2008 (52.79) |  |  |
| **SBP, mmHg, Mean±SD** | 130.12 ± 19.84 | 130.14 ± 19.61 | t=-0.04 | 0.970 |
| **DBP, mmHg, Mean±SD** | 69.61 ± 12.13 | 69.55 ± 11.96 | t=0.21 | 0.835 |
| **Glycohemoglobin, %, Mean±SD** | 5.90 ± 0.96 | 5.90 ± 0.96 | t=-0.02 | 0.985 |
| **TC, mmol/L, Mean±SD** | 5.09 ± 1.14 | 5.09 ± 1.14 | t=0.08 | 0.933 |
| **HDL, mmol/L, Mean±SD** | 1.43 ± 0.44 | 1.43 ± 0.44 | t=-0.00 | 1.000 |
| **ALT/AST, Mean±SD** | 0.92 ± 0.27 | 0.92 ± 0.27 | t=-0.05 | 0.963 |
| **A/G, Mean±SD** | 1.49 ± 0.31 | 1.49 ± 0.31 | t=0.07 | 0.944 |
| **eGFR, Mean±SD** | 76.94 ± 22.81 | 76.97 ± 22.69 | t=-0.06 | 0.953 |
| **UACR, mg/g, M (Q_1_, Q_3_)** | 688.13 (404.38, 1412.58) | 704.95 (408.00, 1496.39) | Z=-0.923 | 0.356 |

PIR, poverty income ratio; GED, general education development; AA, associate; SBP, systolic blood pressure; DBP, diastolic blood pressure; TC, total cholesterol; HDL, high-density lipoprotein; ALT/AST, alanine transaminase/aspartate transaminase; A/G, albumin/globulin; eGFR, estimated glomerular filtration rate; UACR, urinary albumin creatinine ratio; SD, standard deviation; M, median; Q_1_, 1st quartile; Q_3_, 3st quartile.

Supplementary Table 2 Weighted univariate Cox regression analysis to explore potential confounding factors associated with all-cause mortality in OA.

| **Variables** | **HR (95% CI)** | ***P*** |
| --- | --- | --- |
|  |  |  |
| **Age** | 3.75 (3.20-4.41) | <0.001 |
| **Sex** |  |  |
| Female | Ref |  |
| Male | 1.28 (1.06-1.55) | 0.010 |
| **Race** |  |  |
| Non-Hispanic black | Ref |  |
| Non-Hispanic white | 1.04 (0.81-1.34) | 0.765 |
| Other | 0.74 (0.50-1.09) | 0.122 |
| **PIR** |  |  |
| Low/median | Ref |  |
| High | 0.54 (0.44-0.66) | <0.001 |
| **Marital status** |  |  |
| Married/living with partner | Ref |  |
| Divorced/widowed/separated | 2.14 (1.70-2.69) | <0.001 |
| Spinsterhood | 0.86 (0.55-1.36) | 0.530 |
| **Education** |  |  |
| Below high school | Ref |  |
| High school/GED | 0.59 (0.46-0.76) | <0.001 |
| College and above | 0.42 (0.32-0.55) | <0.001 |
| Some college/AA degree | 0.58 (0.46-0.73) | <0.001 |
| **Smoking** |  |  |
| No | Ref |  |
| Yes | 1.28 (1.05-1.57) | 0.016 |
| **Drinking** |  |  |
| No | Ref |  |
| Yes | 0.78 (0.63-0.96) | 0.021 |
| Unknown | 1.24 (0.75-2.03) | 0.398 |
| **Hypertension** |  |  |
| No | Ref |  |
| Yes | 3.17 (2.50-4.02) | <0.001 |
| **Diabetes** |  |  |
| No | Ref |  |
| Yes | 1.77 (1.46-2.15) | <0.001 |
| **Dyslipidemia** |  |  |
| No | Ref |  |
| Yes | 1.15 (0.94-1.40) | 0.180 |
| **CVD** |  |  |
| No | Ref |  |
| Yes | 2.66 (2.18-3.23) | <0.001 |
| **COPD** |  |  |
| No | Ref |  |
| Yes | 1.12 (0.96-1.32) | 0.156 |
| **Depression** |  |  |
| No | Ref |  |
| Yes | 1.48 (1.18-1.86) | <0.001 |
| Unknown | 1.41 (1.10-1.81) | 0.006 |
| **Cancer** |  |  |
| No | Ref |  |
| Yes | 1.80 (1.49-2.17) | <0.001 |
| **CKD** |  |  |
| No | Ref |  |
| Yes | 1.53 (1.24-1.88) | <0.001 |
| **Drug therapy** |  |  |
| No | Ref |  |
| Yes | 0.81 (0.61-1.09) | 0.169 |
| **BMI** |  |  |
| <30 | Ref |  |
| ≥30 | 0.83 (0.69-1.01) | 0.065 |
| **Physical activity** |  |  |
| Mild | Ref |  |
| Median/Strenuous | 0.50 (0.40-0.62) | <0.001 |
| Unknown | 1.35 (1.03-1.77) | 0.029 |
| **ALT/AST** | 0.55 (0.47-0.65) | <0.001 |
| **A/G** | 0.70 (0.61-0.80) | <0.001 |
| **NLR** | 1.32 (1.24-1.41) | <0.001 |

OA, osteoarthritis; PIR, poverty income ratio; GED, general education development; AA, associate; CVD, cardiovascular disease; COPD, obstructive pulmonary disease; CKD, chronic kidney disease; ALT/AST, alanine transaminase/aspartate transaminase; A/G, albumin/globulin; NLR, neutrophil-to-lymphocyte ratio; HR, hazard ratio; CI, confidence level; Ref, reference.

Supplementary Table 3 Weighted multivariate Cox regression analysis to determine final covariates.

| **Variables** | **HR (95% CI)** | *P* |
| --- | --- | --- |
| **Age** | 3.00 (2.62-3.42) | <0.001 |
| **Sex** |  |  |
| Female | Ref |  |
| Male | 2.01 (1.67-2.42) | <0.001 |
| **Marital status** |  |  |
| Married/living with partner | Ref |  |
| Divorced/widowed/separated | 1.47 (1.23-1.77) | 0.001 |
| Spinsterhood | 1.80 (1.24-2.62) | 0.006 |
| **Smoking** |  |  |
| No | Ref |  |
| Yes | 1.35 (1.14-1.60) | 0.004 |
| **Hypertension** |  |  |
| No | Ref |  |
| Yes | 1.61 (1.27-2.04) | <0.001 |
| **CVD** |  |  |
| No | Ref |  |
| Yes | 1.29 (1.08-1.53) | 0.009 |
| **Depression** |  |  |
| No | Ref |  |
| Yes | 1.72 (1.42-2.07) | <0.001 |
| Unknown | 1.47 (1.15-1.88) | 0.003 |
| **CKD** |  |  |
| No | Ref |  |
| Yes | 1.64 (1.23-2.21) | <0.001 |
| **ALT/AST** | 0.75 (0.67-0.84) | 0.001 |
| **A/G** | 0.75 (0.68-0.82) | <0.001 |
| **NLR** | 1.21 (1.14-1.28) | <0.001 |

CVD, cardiovascular disease; CKD, chronic kidney disease; ALT/AST, alanine transaminase/aspartate transaminase; A/G, albumin/globulin; NLR, neutrophil-to-lymphocyte ratio; HR, hazard ratio; CI, confidence level; Ref, reference.
